# Development and validation of a competitive risk model in patients with rectal cancer: based on SEER database

**DOI:** 10.1186/s40001-023-01357-3

**Published:** 2023-09-21

**Authors:** Ruobing Hu, Xiuling Li, Xiaomin Zhou, Songze Ding

**Affiliations:** 1 Department of Gastroenterology and Hepatology, People’s Hospital of Zhengzhou University, No.7 Weiwu Road, Zhengzhou, 450003 Henan China; 2 Department of Infection Disease, Shanghai Jinshan District Tinglin Hospital, Shanghai, 201505 China

**Keywords:** Validation, Competitive risk model, Rectal cancer, SEER database

## Abstract

**Background:**

Rectal cancer is one of the most common malignancies. To predict the specific mortality risk of rectal cancer patients, we constructed a predictive nomogram based on a competing risk model.

**Methods:**

The information on rectal cancer patients was extracted from the SEER database. Traditional survival analysis and specific death analysis were performed separately on the data.

**Results:**

The present study included 23,680 patients, with 16,580 in the training set and 7100 in the validation set. The specific mortality rate calculated by the competing risk model was lower than that of the traditional survival analysis. Age, Marriage, Race, Sex, ICD-O-3Hist/Behav, Grade, AJCC stage, T stage, N stage, Surgery, Examined LN, RX SUMM-SURG OTH, Chemotherapy, CEA, Deposits, Regional nodes positive, Brain, Bone, Liver, Lung, Tumor size, and Malignant were independent influencing factors of specific death. The overall C statistic of the model in the training set was 0.821 (Se = 0.001), and the areas under the ROC curve for cancer-specific survival (CSS) at 1, 3, and 5 years were 0.842, 0.830, and 0.812, respectively. The overall C statistic of the model in the validation set was 0.829 (Se = 0.002), and the areas under the ROC curve for CSS at 1, 3, and 5 years were 0.851, 0.836, and 0.813, respectively.

**Conclusions:**

The predictive nomogram based on a competing risk model for time-specific mortality in patients with rectal cancer has very desirable accuracy. Thus, the application of the predictive nomogram in clinical practice can help physicians make clinical decisions and follow-up strategies.

## Background

Rectal cancer (RC) is a type of cancer that occurs at the dentate line and colon-sigmoideum junctions [1]. Colorectal cancer is the fourth deadliest cancer in the world [2], and RC represents 40% of colorectal cancer cases. In the United States, there are 44,850 new RC cases (26,650 in men; 18,200 in women) in 2022 and 46,050 new RC cases (27,440 in men, 18,610 in women) in 2023, suggesting an increasing incidence [3]. Furthermore, the morbidity and mortality of RC are projected to rise dramatically by 2035 [4]. This cancer may be caused by several controllable risk factors, such as sedentary work [OR = 1.08 (95%CI: 1.00–1.16)] [5], male obesity [RR = 1.12 (95%CI: 1.09–1.16)] [6], red meat intake [RR = 1.22 (95% CI: 1.01–1.46)] [7], meat products [RR = 1.26 (95% CI = 1.09–1.45)] [7], and high alcohol intake [OR = 1.25 (95%CI: 1.11–1.40)] [8].

The distal third of the rectum is drained by systemic veins instead of the portal venous system, which increases the likelihood of direct lung metastases [9]. RC penetrates the pelvis more easily than colon cancer, with a higher risk of lymph node metastasis in each T stage, complex anatomical structures, difficult-to-operate surgery, and a high postoperative recurrence rate. Currently, treatments for RC include local or extensive surgery, preoperative radiotherapy and systemic therapy, local ablation of metastases, palliative chemotherapy, targeted therapy, and immunotherapy [10]. The selection of treatments is based on the tumor’s clinicopathological characteristics, such as the degree of differentiation and the site of metastasis [11, 12]. RC survival is closely related to clinical decisions based on staging information. The 5-year disease-free survival (DFS) and overall survival (OS) of patients with RC were 70.1% (95%CI: 60.7%–73.0%) and 76.2% (95%CI: 73.8%–78.5%) with open surgery, and 72.2% (95%CI: 69.4%–74.8%) and 72.7% (95%CI: 69.8%–75.3%) with laparoscopic surgery, respectively [13]. Compared with long-course chemoradiotherapy, both total neoadjuvant therapy with long-course chemoradiotherapy [OR = 1.78 (95%CI: 1.43–2.26)] and total neoadjuvant therapy with short-course radiotherapy [OR = 1.75(95%CI: 1.23–2.50)] improved the pathological complete response rate; neoadjuvant chemotherapy increased DFS, but did not significantly prolong OS [14]. At present, TNM staging is the primary reference in clinical practice. Other potential prognostic factors, such as age, sex, marriage, and region, are also strongly associated with prognosis [15]. Therefore, it is necessary to explore a more comprehensive prediction method. Clinical predictive models are more personalized approaches tailored to these factors.

The specific mortality risk is more valuable than the traditional mortality risk in clinical practice due to some unforeseeable mortality events. Specifically speaking, the causes of death in RC patients are diverse, and a large number of mortality events are not caused by RC, as RC patients are often in poor functional status or accompanied by co-morbidities [16]. These deaths are unforeseeable when patients die of other diseases, such as cardiovascular disease. In addition, as competing risks often occur among the diverse risk factors for death in patients with RC, traditional survival analysis methods may ignore the occurrence of main concern events, resulting in overestimated results [17]. Therefore, traditional survival analysis methods are unsuitable for processing data containing competing outcomes. A competing risk model is more suitable for dealing with data containing competing events and can produce more accurate results [17].

Since most patients with RC have a long survival period, identifying the risk of specific death early, in the case of competing events, is necessary to facilitate the development of specific prognostic rehabilitation programs and follow-up plans, thereby reducing the risk of specific death in patients. Although various prognostic models, especially based on radiomics, have been established in recent years, predictive models in clinical practice should be highly interpretable and applicable. Therefore, we conducted this study based on the SEER database to construct an interpretable nomogram for early predicting the risk of RC-specific mortality.

## Methods

### Data sources and extractions

The SEER database, which comprises 18 cancer registries, is the National Cancer Institute's collection of demographic statistics on all cancers diagnosed in representative geographic regions and subpopulations. We collected patient clinicopathological data, demographic information, clinicopathological information, and follow-up data from the database. The patient data were de-identified because the SEER database is publicly available. In addition, ethical approval and informed consent are not required for our study. Our methodology strictly follows the rules of the SEER database.

We selected the “Incidence SEER Research Plus Data, 18 Registries, Nov 2020 Sub (2000–2018)” database from 2010 to 2015. According to the disease name of RC, we collected the clinicopathological information of all RC patients, including Patient ID, Age, Marriage, Race, Sex, Primary Site- labeled, ICD-O-3Hist/Behav, Grade, Laterality, AJCC stage, AJCC Tstage, AJCC N stage, AJCC M stage, Surgery, Examined LN, RX SUMM-SURG OTH REG, Radiation recode, Chemotherapy, Preoperative CEA, Pleural Effusion Recode, Tumor deposits, Regional nodes positive, Brain, Bone, Liver, Lung, CS Tumor size, Malignant tumor numbers, cause of death classification, Survival months, COD to site rec KM. Exclusion criteria were as follows: (1) unclear modeling variables NA, Blanks, unknown TNM stage (TX, NX); (2) unknown tumor size; (3) unknown surgical method; (4) survival time < 1 month or unknown (5) Age ≤ 18.

### Construction and verification of competitive risk model

We developed a competing risk model by defining deaths from unknown causes or survival cases in RC as censoring events, deaths from specific causes as interest events, and deaths from other causes as competing events. Before building the model, we randomly split the data by 70% and 30% into training and validation sets. A uniform random number distributed between 0 and 1 was assigned to each case, and then these cases were ranked according to their random number. The cases with a random number < 70 percentile were assigned to the training set, and the rest of the cases were assigned to the validation set. Univariate and multivariate analyses were performed in the training set to screen the independent influencing factors of RC, and RC-specific death nomograms were constructed according to the independent influencing factors. The concordance index (C-index) and the area under the receiver operating curve (AUC) were used to measure the accuracy of the prediction nomogram, and the calibration curve was used to measure the calibration degree of the prediction nomogram.

### Statistical analysis

Continuous variables were tested for normal distribution using Shapiro–Wilk test. Normal distributed continuous variables were expressed as mean ± standard deviation (SD), and an independent samples t-test was used to compare the means of the two groups. For continuous variables with skewed distribution, the Mann–Whitney *U* test was performed for comparisons between groups. Count data were expressed as frequency (%); and the chi-square test was used for comparison between multiple groups. A *P* < 0.05 indicated a statistically significant difference. R 4.2.0 was used for modeling, while SPSS 2.3.0 was used for statistical analysis.

## Results

### Patient characteristics

A total of 23,680 RC patients were included in the analysis. Patients were randomly assigned to the training cohort (*n* = 16,580) and the validation cohort (*n* = 7100). In the training cohort, the mean age of patients was 63.02 ± 13.38 years; the number of deaths was 6470; the number of specific deaths was 4602; and the mean follow-up time was 52.35 ± 27.20 years. In the validation cohort, the mean age of patients was 62.90 ± 13.41 years; the number of deaths was 9174; the number of specific deaths was 6537; and the mean follow-up time was 52.53 ± 27.77 years. It can be seen that the distribution of the two groups is even, and the rest of the information is shown in Table 1.

**Table 1:** Clinicopathological characteristics of RC patients

Factors	Define	Train (*N* = 16,580)	Test (*N* = 7100)	All (*N* = 23,680)	Statistic	*P*
Age (years)		63.02 ± 13.38	62.90 ± 13.41	62.98 ± 13.39	0.652	0.514
Time (years)		52.35 ± 27.20	52.53 ± 27.77	52.41 ± 27.93	− 0.456	0.648
Marriage	Married	9830 (59.3)	4192 (59)	14,022 (59.2)	0.81	0.981
	Single	2827 (17.1)	1211 (17.1)	4038 (17.1)		
	Widowed	1902 (11.5)	825 (11.6)	2727 (11.5)		
	Other	2021 (12.2)	872 (12.3)	2893 (12.2)		
Race	White	13,513 (81.5)	5787 (81.5)	19,300 (81.5)	2.516	0.284
	Black	1416 (8.5)	572 (8.1)	1988 (8.4)		
	Other	1651 (10)	741 (10.4)	2392 (10.1)		
Sex	Female	6649 (40.1)	2869 (40.4)	9518 (40.2)	0.194	0.66
	Male	9931 (59.9)	4231 (59.6)	14,162 (59.8)		
Deposits	Yes	970 (5.9)	423 (6)	1393 (5.9)	0.449	0.799
	No	11,545 (69.6)	4913 (69.2)	16,458 (69.5)		
	Others	4065 (24.5)	1764 (24.8)	5829 (24.6)		
Behav	Behav1	11,851 (71.5)	5029 (70.8)	16,880 (71.3)	1.973	0.578
	Behav2	1383 (8.3)	592 (8.3)	1975 (8.3)		
	Behav3	1630 (9.8)	739 (10.4)	2369 (10)		
	Other	1716 (10.3)	740 (10.4)	2456 (10.4)		
Grade	I	1457 (8.8)	622 (8.8)	2079 (8.8)	0.073	0.964
	II	12,812 (77.3)	5479 (77.2)	18,291 (77.2)		
	III、IV	2311 (13.9)	999 (14.1)	3310 (14)		
AJCC Stage	I	4803 (29)	2106 (29.7)	6909 (29.2)	2.562	0.464
	II	4096 (24.7)	1692 (23.8)	5788 (24.4)		
	III	5849 (35.3)	2526 (35.6)	8375 (35.4)		
	IV	1832 (11)	776 (10.9)	2608 (11)		
T Stage	T1	3250 (19.6)	1468 (20.7)	4718 (19.9)	4.120	0.249
	T2	2882 (17.4)	1191 (16.8)	4073 (17.2)		
	T3	8905 (53.7)	3784 (53.3)	12,689 (53.6)		
	T4	1543 (9.3)	657 (9.3)	2200 (9.3)		
N Stage	N0/N1	14,733 (88.9)	6274 (88.4)	21,007 (88.7)	1.211	0.271
	N2	1847 (11.1)	826 (11.6)	2673 (11.3)		
M Stage	M0	14,748 (89)	6324 (89.1)	21,072 (89)	0.073	0.787
	M1	1832 (11)	776 (10.9)	2608 (11)		
Surgery	No	2291 (13.8)	1011 (14.2)	3302 (13.9)	0.736	0.391
	Yes	14,289 (86.2)	6089 (85.8)	20,378 (86.1)		
LNSur	No	4572 (27.6)	1990 (28)	6562 (27.7)	0.681	0.711
	Yes	11,853 (71.5)	5040 (71)	16,893 (71.3)		
	Unknown	155 (0.9)	70 (1)	225 (1)		
Radiation	Yes	9618 (58)	4143 (58.4)	13,761 (58.1)	0.240	0.625
	No	6962 (42)	2957 (41.6)	9919 (41.9)		
Chemotherapy	Yes	10,739 (64.8)	4649 (65.5)	15,388 (65)	1.095	0.295
	No	5841 (35.2)	2451 (34.5)	8292 (35)		
CEA	Yes	4492 (27.1)	1966 (27.7)	6458 (27.3)	0.993	0.609
	No	5911 (35.7)	2524 (35.5)	8435 (35.6)		
	Others	6177 (37.3)	2610 (36.8)	8787 (37.1)		
Brain	No	16,559 (99.9)	7096 (99.9)	23,655 (99.9)	2.331	0.127
	Yes	21 (0.1)	4 (0.1)	25 (0.1)		
Positive	No	7889 (47.6)	3370 (47.5)	11,259 (47.5)	0.486	0.784
	Yes	4184 (25.2)	1771 (24.9)	5955 (25.1)		
	Other	4507 (27.2)	1959 (27.6)	6466 (27.3)		
Liver	No	15,320 (92.4)	6578 (92.6)	21,898 (92.5)	0.437	0.508
	Yes	1260 (7.6)	522 (7.4)	1782 (7.5)		
Lung	No	16,031 (96.7)	6862 (96.6)	22,893 (96.7)	0.026	0.872
	Yes	549 (3.3)	238 (3.4)	787 (3.3)		
Bone	No	16,476 (99.4)	7064 (99.5)	23,540 (99.4)	1.222	0.269
	Yes	104 (0.6)	36 (0.5)	140 (0.6)		
Size(cm)	< 3	5124 (30.9)	2215 (31.2)	7339 (31)	0.278	0.87
	3–5	5304 (32)	2250 (31.7)	7554 (31.9)		
	5–	6152 (37.1)	2635 (37.1)	8787 (37.1)		
Malignant	1	12,405 (74.8)	5379 (75.8)	17,784 (75.1)	2.440	0.295
	2	3224 (19.4)	1335 (18.8)	4559 (19.3)		
	3–	951 (5.7)	386 (5.4)	1337 (5.6)		
Status	0	10,130 (61.1)	4376 (61.6)	14,506 (61.3)	0.693	0.707
	1	4602 (27.8)	1935 (27.3)	6537 (27.6)		
	2	1848 (11.1)	789 (30)	2637 (11.1)		

Deposits, Tumor deposits; Behav, Pathological type; Grade, differentiation Grade; AJCC, American Joint Committee on Cancer; LNSur, regional Lymph Node Surgery Information; CEA, Carcinoembryonic antigen; Brain, brain metastasis; Positive, positive lymph nodes; Liver, metastasis; Lung, Lung metastasis; Bone, bone metastasis; Malignant, the number of malignant tumors. Status, 0 (Alive or dead of other cause) & 1 (Dead attributable to RC cancer) & 2 (Unknown)

### Competing risk model for univariate and multivariate analysis

The variables in the present study included Age, Marriage, Race, Sex, ICD-O-3Hist/Behav, Grade, AJCC stage, T stage, N stage, Surgery, Examined LN, RX SUMM-SURG OTH, Chemotherapy, CEA, Deposits, Regional nodes in single factor Positive, Brain, Bone, Liver, Lung, Tumor size, and Malignant variables. Both univariate and multivariate analyses did not include the M stage because it overlapped with the AJCC stage. The variable Radiation (*P* > 0.05) was excluded from the multivariate analysis, whereas other variables in the single factor analysis were included in the multivariate analysis, with *P* < 0.05 (Table 2). The results showed that these variables were all independent influencing factors for RC. Accordingly, we constructed a competing risk model based on these variables.

**Table 2:** single factor and multi factor analysis

Factors	Define	Univariate analysis		Multivariate analysis	
		HR (95%CI)	*Z* (*P*)	HR (95%CI)	*Z* (*P*)
Age		1.014 (1.011–1.016)	11.255 (< 0.001)	1.016 (1.013–1.019)	10.966 (0)
Marriage	Married	Ref.			
	Single	1.354 (1.254–1.462)	7.723 (< 0.001)	1.233 (1.133–1.341)	4.854 (0)
	Widowed	1.576 (1.443–1.721)	10.146 (< 0.001)	1.317 (1.188–1.461)	5.236 (0)
	Other	1.275 (1.167–1.392)	5.384 (< 0.001)	1.174 (1.064–1.297)	3.178 (0.002)
Race	White	Ref.			
	Black	1.341 (1.220–1.474)	6.084 (< 0.001)	1.205 (1.086–1.336)	3.532 (0)
	Other	1.026 (0.931–1.130)	0.512 ( > 0.05)	1.114 (1.009–1.23)	2.145 (0.032)
Sex	Female	Ref.			
	Male	1.105 (1.041–1.173)	3.300 (< 0.001)	1.161 (1.085–1.241)	4.355 (0)
Deposits	Yes	Ref.			
	No	0.371 (0.335–0.410)	− 19.456 (< 0.001)	0.585 (0.526–0.651)	− 9.814 (0)
	Others	1.062 (0.958–1.178)	1.150 ( > 0.05)	0.802 (0.706–0.912)	− 3.359 (0.001)
Behav	Behav1	Ref.			
	Behav2	0.508 (0.445–0.580)	− 9.973 (*P* < 0.001)	0.856 (0.745–0.984)	− 2.191 (0.028)
	Behav3	0.495 (0.437–0.561)	− 11.061 (*P* < 0.001)	0.91 (0.796–1.039)	− 1.392 (0.16)
	Other	1.223 (1.119–1.336)	4.459 (*P* < 0.001)	1.059 (0.952–1.177)	1.052(0.29)
Grade	I	Ref.			
	II	1.420 (1.257–1.604)	5.630 (*P* < 0.001)	1.214 (1.069–1.379)	2.991 (0.003)
	III, IV	2.615 (2.288–2.990)	14.070 (*P* < 0.001)	1.641 (1.419–1.898)	6.682 (0)
AJCC Stage	I	Ref.			
	II	2.123 (1.914–2.354)	14.258 (*P* < 0.001)	1.69 (1.437–1.988)	6.332 (0)
	III	2.840 (2.583–3.122)	21.607 (*P* < 0.001)	1.735 (1.479–2.035)	6.766 (0)
	IV	11.515 (10.421–12.723)	47.997 (*P* < 0.001)	3.658 (2.968–4.507)	12.169 (0)
T	T1	Ref.			
	T2	0.926 (0.817–1.050)	− 1.110 (*P* > 0.05)	0.931 (0.814–1.066)	− 1.037 (0.3)
	T3	2.046 (1.864–2.246)	15.069 (*P* < 0.001)	1.138 (0.994–1.303)	1.867 (0.062)
	T4	4.487 (4.019–5.011)	26.678 (*P* < 0.001)	1.764 (1.512–2.059)	7.201 (0)
N	N0/N1	Ref.			
	N2	2.336 ( 2.173–2.510)	23.080 (*P* < 0.001)	1.261 (1.153–1.379)	5.073 (0)
Surgery	1	Ref.			
	2	0.263 (0.246–0.281)	− 38.849 (*P* < 0.001)	0.47 (0.405–0.546)	− 9.918 (0)
LNSur	No	Ref.			
	Yes	0.584 (0.550–0.621)	− 17.110 (*P* < 0.001)	1.074 (0.884–1.304)	0.72 (0.47)
	Unknown	0.770 (0.588–1.010)	− 1.884 (*P* > 0.05)	1.055 (0.766–1.453)	0.326 (0.74)
Radiation	Yes	Ref.			
	No	1.001 (0.944–1.062)	0.050 (*P* > 0.05)	-	
Chemotherapy	Yes	Ref.			
	No	0.727 (0.682–0.776)	− 9.649 (*P* < 0.001)	1.392 (1.273–1.521)	7.265 (0)
CEA	Yes	Ref.			
	No	0.422 (0.392–0.454)	− 23.330 (*P* < 0.001)	0.724 (0.668–0.784)	− 7.934 (0)
	Others	0.538 (0.503– 0.576)	− 18.013 (*P* < 0.001)	0.858 (0.794–0.927)	− 3.881 (0)
Brain	No	Ref.			
	Yes	8.645 (5.075–14.726)	7.937 (*P* < 0.001)	2.613 (1.727–3.955)	4.545 (0)
Positive	No	Ref.			
	Yes	2.748 (2.560–2.950)	28.004 (*P* < 0.001)	1.685 (1.53–1.856)	10.603 (0)
	Other	2.622 (2.437–2.820)	25.901 (*P* < 0.001)	1.239 (1.025–1.496)	2.221 (0.026)
Liver	No	Ref.			
	Yes	5.424 (5.044–5.833)	45.580 (*P* < 0.001)	1.405 (1.215–1.624)	4.593 (0)
Lung	No	Ref.			
	Yes	5.795 (5.253–6.392)	35.117 (*P* < 0.001)	1.294 (1.121–1.494)	3.511 (0)
Bone	No	Ref.			
	Yes	9.253 (7.282–11.757)	19.049 (*P* < 0.001)	1.712 (1.314–2.232)	3.977 (0)
Size	< 3 cm	Ref.			
	3–5 cm	1.680 (1.546–1.826)	12.242 (*P* < 0.001)	1.089 (0.993–1.196)	1.803 (0.071)
	5–cm	2.579 (2.387–2.786)	24.014 (*P* < 0.001)	1.256 (1.145–1.378)	4.845 (0)
Malignant	1	Ref.			
	2	1.057 (0.983–1.136)	1.498 (P > 0.05)	1.053 (0.97–1.142)	1.226 (0.22)
	3–	1.211 (1.078–1.359)	3.233 (P < 0.05)	1.225 (1.076–1.395)	3.074 (0.002)

HR, Hazard radio; CI, Confidence interval; Deposits, tumor deposits; Behav, pathological type; Grade, differentiation grade; AJCC, American Joint Committee on Cancer; LNSur, regional lymph node surgery information; CEA, carcinoembryonic antigen; Brain, brain metastasis; Positive, positive lymph nodes; Liver, metastasis; Lung, Lung metastasis; Bone, bone metastasis; Malignant, malignant number tumors

### Validation of predictive nomogram

**Fig. 1:**
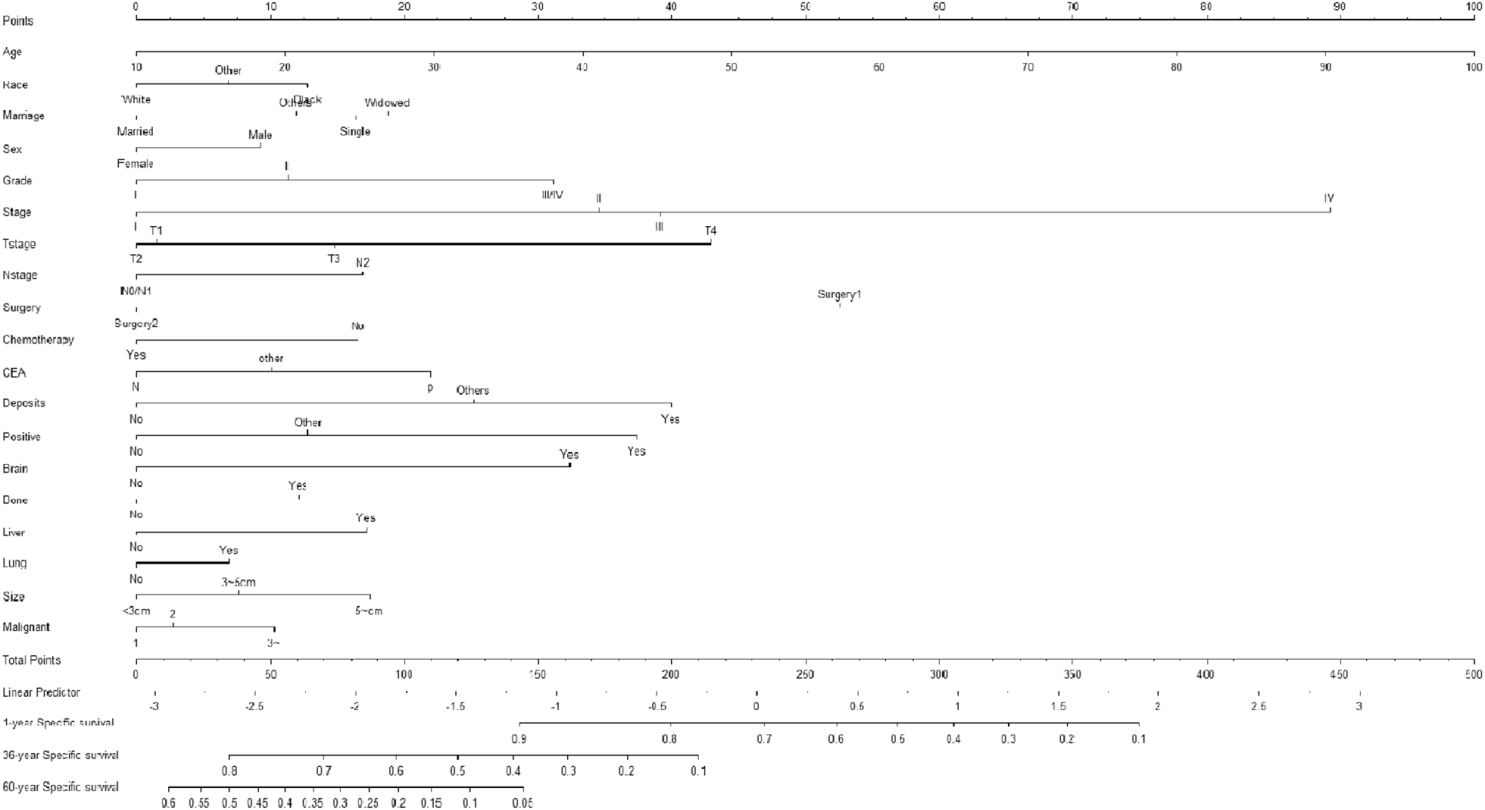
The competitive risk model nomogram of patients with Rectal Cancer at 1, 3, and 5 years

**Fig. 2:**
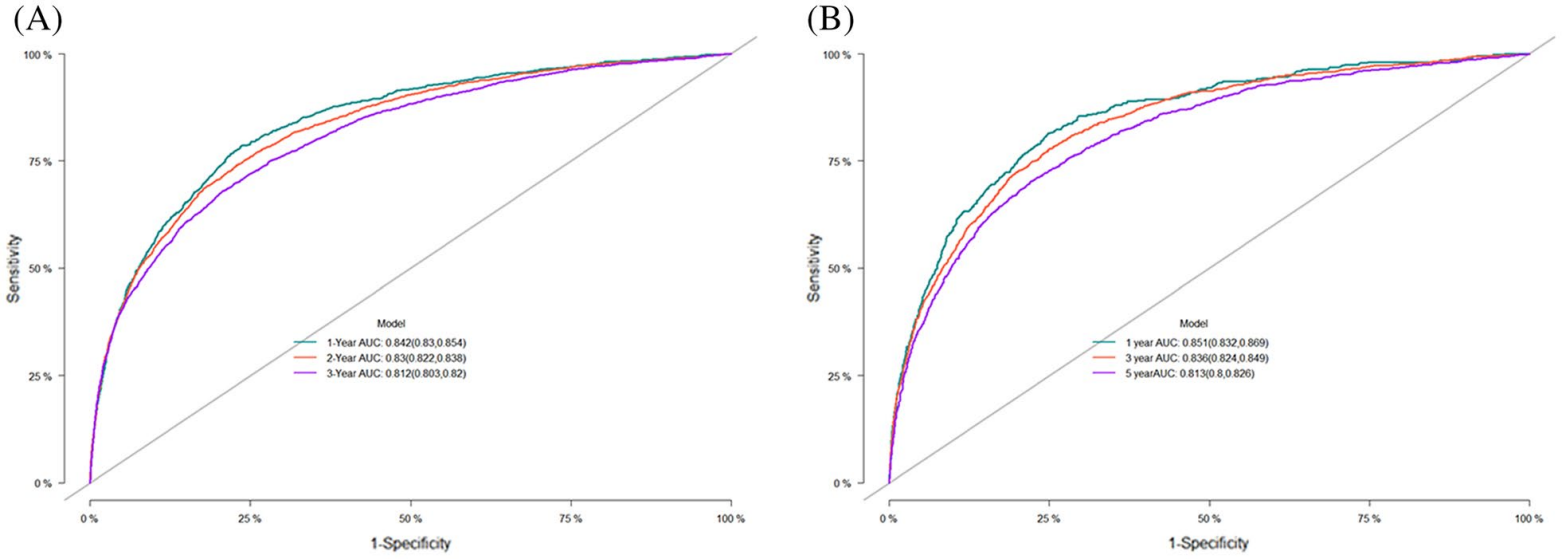
AUC for predicting 1-, 3-, and 5-year Rectal Cancer in training cohort (**A**) and validation cohort (**B**)

**Fig. 3:**
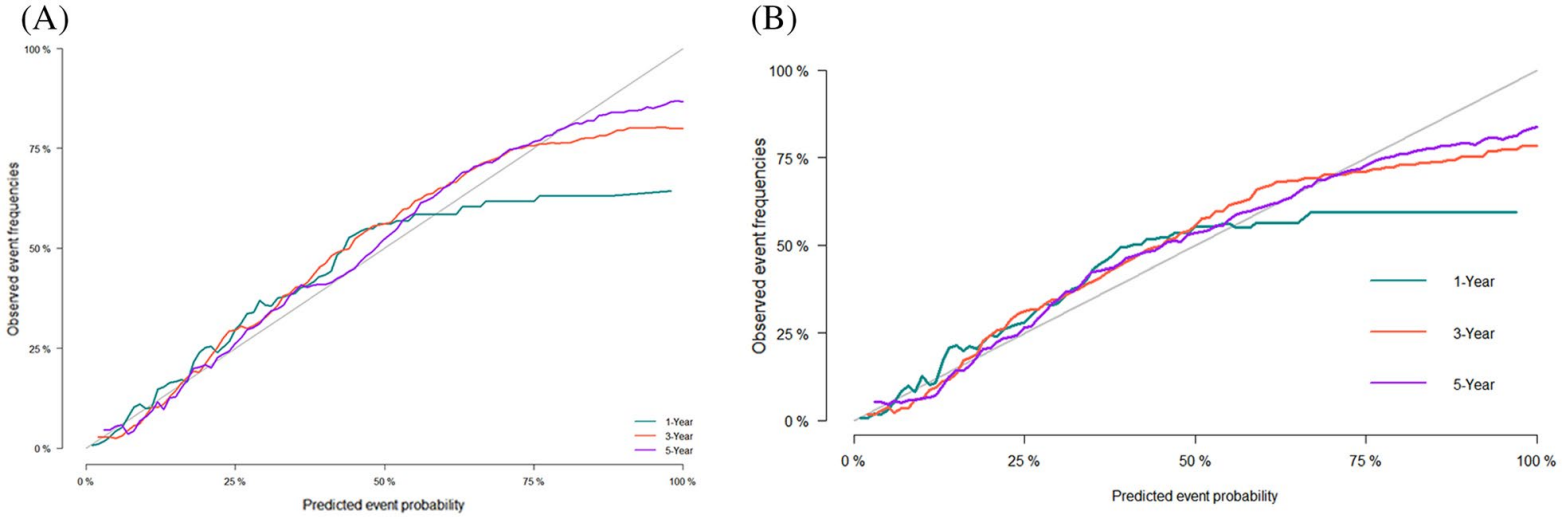
Calibration curve of the nomogram in training cohort (**A**) and validation cohort (**B**)

Specific mortality prediction nomograms were drawn according to the competing risk model described above (Fig. 1). In the training set, the overall C statistic was 0.821 (Se 0.001), and the 1-, 3-, and 5-year AUC for predicting the specific mortality was 0.842 (95% CI 0.830–0.854), 0.830 (95% CI 0.822–0.838), 0.812 (95% CI 0.803–0.820), respectively. The results show that the model has a good identification ability (Fig. 2A). In addition, the model's calibration curve (Fig. 3A) shows that the 1-, 3- and 5-year curves are consistent with the ideal curve, indicating that the predicted curve diagram has good accuracy and calibration.

At the same time, in the internal validation cohort, the overall C statistic was 0.829 (Se 0.002); the 1-, 3-, and 5-year AUC (Fig. 2B) was 0.851 (95% CI 0.832–0.869), 0.836 (95% CI 0.824–0.849), and 0.813 (95% CI 0.800–0.826). The results also show that the model has good identification power. According to the calibration curve of the model (Fig. 3B), the 1-, 3- and 5-year curves are consistent with the ideal curve, indicating that the predicted curve diagram has good accuracy and calibration.

### Specific mortality estimation

We compared the 1- to 9-year specific mortality calculated by traditional survival analysis and the competing risk model. The results revealed that the competing risk model showed lower specific mortality than traditional survival analysis. For example, the 1-year mortality rate was 0.08 in the traditional survival analysis and 0.07 in the specific survival analysis. Their gap grows as the survival duration increases between 1 and 9 years. Therefore, mortality from other causes could not be ignored (Table 3).

**Table 3:** Comparison of 1- to 9-year specific mortality (%) between traditional survival analysis and competitive risk model

Time (year)	Traditional survival analysis	Competitive risk model	
		Specific mortality	Mortality from other causes
1	0.08	0.07	0.02
2	0.15	0.13	0.05
3	0.21	0.18	0.06
4	0.26	0.23	0.08
5	0.3	0.27	0.10
6	0.33	0.29	0.11
7	0.36	0.32	0.13
8	0.38	0.33	0.15

## Discussion

Based on univariate and multivariate analyses, we included the independent influencing factors of RC-specific death, including Age, Marriage, Race, Sex, ICD-O-3Hist/Behav, Grade, AJCC stage, T stage, N stage, Surgery, Examined LN, RX SUMM-SURG OTH, Chemotherapy, CEA, Deposits, Regional nodes positive, Brain, Bone, Liver, Lung, Tumor size, and Malignant. These variables were combined to construct a competing risk model for RC and a predictive nomogram, which have excellent accuracy. The overall C statistic was 0.821 (Se 0.001) in the model training cohort and 0.829 (Se 0.002) in the internal validation cohort.

Currently, there are various RC prediction nomograms based on clinical data [18–21]. However, they are based on Cox regression. For example, a previous study reported that the consistency index (C index) was 0.71 (95% CI: 0.64–0.79) in the training cohort and 0.69 (95% CI: 0.61–0.78) in the validation cohort [21]. According to another research that constructed an RC risk prediction model based on the SEER database, the C index of the RC model was 0.756 (95% CI, 0.726–0.786) for the internal validation and 0.729 (95% CI, 0.678–0.780) for the external validation [22]. In contrast, the overall C statistic for the model in the validation set in our study was 0.829 (Se = 0.002). The reason for the differences in C-index is that the former two do not consider the effects of other causes of death, which can result in a severe bias in the specific mortality and an overestimated specific mortality. In our study, deaths from other causes accounted for approximately 10%. If these deaths were ignored, the accuracy of the model would be reduced. We compared the results of the competing risk model for RC and the traditional Cox proportional hazards model, and found differences in their mortality estimates. Other studies also have reported similar results and proposed that the COX model may also misestimate the direction of independent risk factors and the correlation between outcomes [23]. According to another competing risk model for early-stage RC-specific mortality [24], the AUC for predicting the 3-, 5-, and 10-year cancer-specific mortality was 82.2, 78.4, and 75 in the training cohort, and 83.4, 75.9, and 76.8 in the validation cohort, respectively. The different results may be due to an early-stage population, a small number of included cases, and insufficient risk indicators in the study.

Currently, the most widely used tumor prognosis prediction tool is the TNM staging. However, the use of the TNM system for RC has been questioned in recent years [25]. In some studies, a series of oxidative stress indicators significantly correlated with the survival rate of colorectal cancer patients were included to establish a CIOSS score system. The CIOSS score was reported to outperform the TNM staging in terms of survival prediction in patients with colorectal cancer [26]. Furthermore, a recent analysis showed that the survival pattern of colorectal cancer was heterogeneous even within the TNM classification [27]. Furthermore, a study attempted to use molecular and biochemical markers to aid in colon cancer staging [28]. In our research, specificity factors were added. Carcinoembryonic antigen (CEA) is now the most widely adopted and readily available tumor marker [29]. Many clinical scientists have evaluated the kinetic patterns of tumor markers as predictors [30]. One study found that patients with elevated postoperative CEA had an increased risk of recurrence [31]. Another study found a direct relationship between tumor volume and overall survival in RC patients [32]. Furthermore, the ratio of serum CEA to maximum tumor diameter may be a more important indicator for assessing tumor bioactivity with higher predictive value in RC [33]. Tumor deposits (TDs), especially solitary tumor nodules (in the mesocolic and mesorectal adipose tissue) at the lymphatic drainage zone of the primary tumor, are a hallmark of RC aggressiveness. The clinical value of TDs in treating RC is severely underestimated [34]. TD-positive tumors are classified as N1c without lymph node metastasis (LNM) in the NCCN guidelines [35], whereas neither the presence of TD nor the number of TD is considered for cases with LNM in the pN staging system. Recently, a clinical study found that tumor deposits in locally advanced RC had great predictive value in RC patients. TDs combined with lymph node metastasis could improve the accuracy of TNM staging [35–37]. In addition, CEA [38, 39] and tumor deposits [40] were strongly associated with RC-specific death based on meta-analyses and evidence-based evidence.

The number of indicators involved in this paper is limited. More indicators should be included in the future to further explore the relationship between the research mechanism, clinical indicators, and prognosis. The occurrence of RC is thought to be closely related to the inflammation and oxidative stress of the intestinal epithelium, damaging the integrity of the intestinal barrier. Exposure to environmental toxins in the intestine enhances intestinal inflammation and releases reactive oxygen species (ROS) [41]. The indicators mentioned in this study, including the primary site of cancer [42], the surgical method [43], and lymph node organ metastasis [44], are all related to oxidative stress. For example, it was found that RC patients with a right-sided primary site had higher mean malondialdehyde serum levels than those with a primary site elsewhere [42]. The serum 8-isoprostanes (8-epiPGF2α) could be used to judge the oxidative state, and the degree of oxidative stress was found to be lower in laparoscopic surgery than in open RC surgery [43]. Additionally, the urinary 8-hydroxydeoxyguanosine (8-OHdG) content in colorectal cancer patients gradually increased from stage I to stage IV, and the urinary 8-OHdG content in patients with tumor metastasis was significantly higher than that of patients without tumor metastasis [42]. More oxidative stress or other specific indicators will be incorporated in the future.

### Limitations

In conclusion, the predictive nomogram of a competitive risk model with competing events is constructed for the first time based on a large number of sample data. There are still some limitations in our study. First, it is insufficient to use a competing risk model and key variables to reflect other causes of death, based on the SEER database. Second, some key predictors of specific mortality risk seem to be missing in the SEER database. Third, our research data are extracted from the SEER database, which includes multiple races. However, there are a large number of missing values in the case registration. When there are too many missing values, it is inappropriate to blindly use the imputation method, and deleting these data is more suitable. However, this may cause some biases, which is an inevitable flaw in the research based on large databases. Fourth, there is a lack of external data validation. Although our study covers the global population, it is still dominated by whites and blacks. There are only a small number of patients from other races, which may cause biased results to some extent. External validation will be the direction of our follow-up research after this model is applied to clinical practice.

### Outlook

As statistical theory and computer systems have advanced considerably over recent years, precision medicine has attracted widespread attention, resulting in the promotion and application of artificial intelligence in clinical practice. With the popularization of image-based artificial intelligence in clinical practice, the application status of machine learning models based on radiomics in rectal cancer has been summarized by researchers. Current intractable challenges lie in diverse sources of radiomics, over-provision equipment, specificity of manual segmentation of regions of interest, and the screening of variables [45, 46].

Furthermore, some additional challenges cannot be ignored, such as data quality, goodness of fit of machine learning, evidence of general applicability, and moral considerations [47]. Despite these challenges, the application of radiomics in clinical practice is still epoch-making, especially for the diagnosis of tumors [47, 48]. In addition, radiomics-based machine learning has gradually been used to predict the prognosis of tumors, such as response to chemotherapy, recurrence, metastasis, and mortality risk. In this context, some studies have applied radiomics to the clinical diagnosis and treatment of RC. A systematic review by Bedrikovetski et al. [49] shows that radiomics is efficient for the identification of lymph node metastasis in colorectal cancer. Di Re et al. [50] and Bourbonne et al. [51] also believe that this technique has relatively ideal predictive value for the response to neoadjuvant chemotherapy in patients with RC or colorectal cancer. In addition, Staal et al. [52] reviewed the performance of radiomics in the prediction of survival outcomes in patients with colorectal cancer; however, they only focused on OS. Although the predictive nomogram we constructed shows a relatively ideal predictive value, its predictive performance still needs to be improved. Subsequent studies are desired to combine radiomics and clinical features to build a more efficient predictive tool for specific death.

## Conclusion

Our predictive nomogram constructed based on a competing risk model is highly accurate in predicting time-specific mortality in RC patients and can assist clinicians in making clinical decisions and developing follow-up strategies. Subsequent studies should incorporate some other key factors or use other radiomics methods to further enhance the predictive performance of the model.

## Data Availability

The data that support the findings of this study are available from the corresponding author upon reasonable request.

## Abbreviations

RC: Rectal cancer
CSS: Cancer-specific survival
CEA: Carcinoembryonic antigen
TDs: Tumor deposits
LNM: Lymph node metastasis
ROS: Reactive oxygen species
